# A Healthcare Pathway to Nirvana? The SNF Transition to Home

**DOI:** 10.3390/geriatrics3030054

**Published:** 2018-08-24

**Authors:** Wayne S. Saltsman

**Affiliations:** Chief Medical Officer, Continuing Care, Lahey Health, Burlington, MA 01803, USA; wayne.s.saltsman@lahey.org; Tel.: +1-781-756-7716

**Keywords:** transitions of care, skilled nursing, advance care planning

## Abstract

While the majority of attention and the literature has focused on transitional models out of the acute care setting, transitions from the post-acute setting—especially from the skilled nursing facility (SNF)—are not well understood. What are the ‘best practices’, or thoughtful considerations, for a successful transition back to home and the community? Facilitation of a smooth and seamless transition relies on the abilities of the SNF and primary care teams, as well as community agencies, to coordinate care in a patient-centered manner together. This article will focus on this specific transition within the healthcare continuum.

Dr. James Lett, a former president of the American Medical Director Association, the Society for post-acute and long-term care medicine (AMDA-PALTC), and a former chair of the society’s transitions of care (TOC) committee, said at a 2015 TOC meeting, “the best transition is the one that never occurred”. What did Dr. Lett mean? Transitions occur constantly in healthcare; in fact, they are fundamental to care within the continuum: There are ‘micro’ transitions that occur between hospital providers’ teams or floors, or between primary care practitioners and specialists, and there are ‘macro’ transitions that occur between institutions, i.e., the hospital, the SNF, and home care and community services agencies. How can we fathom, then, that a transition might not occur in the care of our patients? Consider a transition—any transition—that was so seamless, so smooth, with such optimal communication and education between providers, nurses, facilities, case managers, support staff, patients, and families, that it ‘felt’ to everyone as if care was completely coordinated. To that patient and family, it might actually appear that (aside from geography) the logistics around care had not been interrupted or fragmented: that the semblance of a transition had ‘never occurred’. Can we get to this transition ‘Nirvana’ of patient-centered care? In 2018, the AMDA-PALTC TOC committee continues to contemplate the ‘never occurred’ transition state. What would be the ‘lowest hanging fruit’ in the transitions process from which we could take a leading practice ‘bite’ of improvement? This article will suggest that a good place to start is the SNF setting itself; and more specifically, at the transition point between the SNF and the community, a transition that has not been well-studied, but would embrace a stronger, more invested medical practitioner role, and an actionable, cohesive discharge plan (and summary) to potentiate ongoing care.

While the essence of short-term rehabilitation in the SNF setting focuses on raising patient functional abilities to the highest practicable level via nursing monitoring and assessments, as well as nutritional, social work, and therapeutic interventions, the SNF is more than just a place for these aspects of ‘skilled care’. Another, perhaps even more substantial, function of the SNF remains poorly recognized: It is a unique setting within the continuum of care, in which each patient has the potential to be actively monitored and intervened upon, for up to weeks, in ‘real time’. In turn, the SNF patient can truly become the focal point around which families, ambulatory and hospital providers, and home care and community agencies together may participate as a team in communications, education, and outcomes. In this way, the SNF setting can be a ‘hallmark’ of ‘patient-centered care’. Furthermore, in recognition of this setting’s potential significance in post-acute patient transitions, and in accordance with the Improving Medicare Post-Acute Care Transformation Act of 2014 (IMPACT Act), Medicare has become more intimately involved in trying to understand and improve the patient transition process. For example, it established a technical expert panel (TEP) panel, initiated in September of 2016, to determine ‘transition’ quality measures, in part, for when a patient was discharged from post-acute care to other providers and settings. In turn, as work groups such as the TEP were attempting to understand what distinguished quality in transitions, some higher-level, unanswered questions became evident: what were the natures of the SNF provider role, the discharge processes upon which a facility needed to focus, and the roles and responsibilities of the receiving provider(s) and community agency(ies).

The potential of the SNF remains largely untapped because the actual role of the SNF provider, as described by the Medicare code of federal regulations (CFR), i.e., 42 CFR 483.40 (a) and (b), still remains subjective at best. When should a patient be initially seen? What is the best format for documentation? What is the relationship of the SNF provider to the SNF team? What is an impactful visit? Even with further clarification at the state-regulation level, the basic role of the physician is to provide “personal approval” of an admission recommendation, ‘supervise’ or ‘participate’ in resident care, supervise advanced practitioners, and generate visits that are intended to represent a more ‘active role’ in patient management. This description leaves significant room for variation in the approach to the provider–patient relationship and begs for the establishment of ‘best practices’. As the first step in taking full advantage of what the SNF setting has to offer—as the ‘therapeutic hiatus’ of care that it can be—attending physicians and advanced practitioners need to be the SNF multidisciplinary team leaders: they must become more invested in the overall care of their (assigned) patients and they must actively participate in the creation, enforcement, or modification of their patient’s plans of care. Furthermore, AMDA-PALTC, through its ‘core competencies’, wants to help invigorate this role of the SNF attending physician (and further develop that of the advanced practitioner) as a unique leader and specialist in post-acute care, through further, SNF-specific education and practices. In this regard, a SNF provider’s ability to implement ‘proactive’ plans of care can help to fortify a transition back to the community that is as impactful and long-lasting as possible. 

The AMDA-PALTC position statement regarding the role of the attending physician in the nursing home is perhaps more relevant today than at its introduction in 2003, and the provision of care is only augmented more with the evolving team role and responsibilities of the advanced practitioner [1]. The position statement reflected details around seven basic tenets, including the ‘support of patient discharges and transfers’. It brought to light the concept of the ‘warm hand-off’ between providers, assisting in facility discharges processes, and providing ‘pertinent discharge information’ (i.e., within 30 days). However, the nature of the SNF has evolved over the past 15 years, and the 2003 position statement transition recommendations now appear dated. The SNF of today faces increasing patient acuity (higher case mix index), utilization oversight, and penalties associated with lengths of stay, readmissions, and issues around medications; identifying social determinants of health; understanding accountable care organizations, value-based care, Star ratings, and quality metrics; and improving patient satisfaction and outcomes [2,3]. In order for the optimal transition of the patient from the SNF to the primary provider team and community, the SNF practitioner has to be invested in these patient care issues prior to admission. The SNF practitioner has to develop a relationship with the hospital discharge team to help it understand what is necessary for the SNF team to assume appropriate care, including ‘warm hand-offs’. A best practice may be for a new SNF patient to be seen within 24 h of admission for an assessment and review (i.e., history and physical). The review of medications needs to be purposeful, regular, and impactful throughout the course of treatment, with transparency, education, and communicated considerations for modifications or discontinuations (i.e., de-prescribing) as warranted. Practitioners should be at ‘family meetings’, or multidisciplinary rounds, to help coordinate care. Discharge summaries should be completed in a much more timely manner than recommended by the 2003 AMDA-PALTC position statement and then successfully conveyed to the primary team. Appropriately consulted community agencies should be able to enter facilities, meet and learn about their future patients and their needs, and begin working toward continuing the SNF plan of care for ongoing stability at home. The patient, in the meantime, through all of these efforts, cannot help but recognize that he/she is not only the ‘center’ of the multidisciplinary team, but must also be an active ‘player’ on that team. Gone should be the days in which the SNF patient is merely the (passive) receiver of care: care should be deliberate and purposeful, and a patient (and/or family) should be actively involved and also accountable in that care. Moreover, as Medicare continues to review the potential metrics for a quality transition, it should consider how reimbursement opportunities can support the efforts of a provider who takes an active lead in the SNF multidisciplinary team: Greater investment in patient care should be reflected in greater remuneration options; the system needs to recognize not only the efforts of its providers, but also their need to be compensated for those efforts.

This investment in patient-centered care by the provider and multidisciplinary team also makes the SNF an ideal environment for the opportunity to address the needs, goals, and wishes for all patients, but especially for ‘higher risk’ patients or those identified with life-determining illnesses. In Massachusetts, there is a code of regulations (CMR), 130 CMR 140.031, in the long-term care setting to identify such ‘appropriate’ patients and ‘at the minimum’ provide information for advance care planning (ACP) and palliative care or hospice services. In the short-term setting, with the increasing patient acuity on presentation from the hospital, a standardized process for all patients should be to initiate, document, and communicate ACP discussions in order to: (1) confirm advance directives (i.e., the health care proxy (HCP)), (2) identify those patients for whom a palliative care consult would be beneficial, (3) understand patient (or verified surrogate) desires for resuscitation, rehospitalization, or advanced treatment options via a patient and provider-signed statement of ‘orders for life-sustaining treatments’ (for which some states refer to as a ‘MOLST’ or ‘POLST’), or (4) appreciate those patients for whom end-of-life discussions and hospice referrals might be the best plan of care for their successful transition back to the community. These ACP discussions also provide the opportunity to utilize the reimbursement codes that Medicare has established (i.e., 99,497 and 99,498) to incentivize such discussions; but, regardless of regulation or financial benefit, advance care planning is one of the most valuable provider tools for enhancing transitional (and patient-centered) care from the SNF setting [4].

Numerous models for transitions of care from the acute setting have been introduced over recent years (e.g., Coleman, Naylor, BOOST), and in 2013, a Project RED (Re-Engineered Discharge)-based model was utilized to transition a unique set of patients from the post-acute setting to the community [5]. However, with the realization that clinical considerations around the SNF-to-home transition had been “largely neglected”, in 2016, a consensus panel made up of members from the American Geriatrics Society (AGS), the Society for General Internal Medicine (SGIM), and AMDA-PALTC was convened to recommend ‘best practices’ for that transition [6]. The panel determined a transition ‘process map’ (Figure 1) and made subsequent workflow recommendations using previously researched or vetted ‘hospital-based’ practices as references. The SNF-to-home transition ‘best practices’ subsequently developed highlighted four major issues for which both the facility and the receiving primary practitioner (PCP) needed to be accountable: (1) identifying and documenting the correct PCP; (2) scheduling post-discharge follow-up within seven days of SNF discharge with the assurance and patient promotion of accessibility to the PCP office within that time frame; (3) completing and conveying (legible) SNF discharge summaries with instructions that would be read, followed, and incorporated into the patient medical record, and conducting warm hand-offs between the SNF discharge nurse and the receiving community clinician’s office; and (4) ensuring that the patient received a phone call to assess status 48 h following SNF discharge. The transfer of information and the nature of ‘interoperability’ of the electronic medical record, as determined by the IMPACT ACT, would need to be further determined to optimize the process. The consensus panel felt that the adoption of these ‘best practices’, regardless of the potential initial operational challenges on both the SNF and PCP ends of care (i.e., receiving and cataloguing a facsimile document), would provide the “highest quality care” at this transition point, with the expectation that further burdens on the healthcare system (i.e., financial and rehospitalization stressors) would be mitigated, but quality would be assured and patient (family) satisfaction would be optimized.

The successful transition to home also mandates the coordination of care with community agencies, such as visiting nursing/home care, and/or area agencies on aging (AAA)/aging services access points (ASAPs), as there is evidence that reduced thirty-day readmissions may be associated with a home health visit within a week of SNF discharge [7]. AAA/ASAPs are private, not-for-profit agencies that provide case management and implementation of service plans, and many SNFs have an established relationship with their most local AAA/ASAP for assistance in supporting the transition of more dependent patients back to the community. Some AAA/ASAPs, through the use of transitions programs such as the ‘Coleman model’, have successfully put systems in place in the home to reduce a patient’s risk of readmission as well [8,9]. These agencies may be contacted by SNF case management, evaluate patients prior to discharge, and facilitate SNF team transition plans. In order to optimize the transition and carry out crucial roles towards maintaining patient stability and autonomy, other home care agencies, such as “visiting nursing”, specifically, must receive adequate and appropriate discharge information—with most important being the SNF discharge summary—prior to the first home visit, and SNF teams should be available to agency personnel for clarification of orders or questions regarding patient status or ongoing care issues. Similarly, visiting nursing and PCP offices need to work together to operationalize models of communication for any discussions deemed to be necessary in the care of patients who have transitioned home. The visiting nurse, in typically caring for the more complex/higher acuity patient, needs to be seen and respected as a front-line, skilled care provider in order for the coordination of patient care at home to succeed.

Currently, the vision of the seamless, coordinated pathway for patients from the SNF setting to home is far from that of Nirvana, but as one surpasses the many ‘needs’ discussed here for optimal transitional care, the pathway does become clearer. As long as disparities in the working relationships between providers, SNFs, and community teams continue, however, the ‘transition that never occurred’ will not become an actuality. Patient-centered care, at this transition point, demands more than what is ‘regulated’: it requires a true investment in advance care planning and the desire to utilize the SNF as a location—and as an opportunity—to elicit positive change through ongoing communication, education, and commitment to the multi- and interdisciplinary teams. These efforts will yield outcomes in quality that far outweigh those of ‘lengths of stay’ or ‘readmissions’—perhaps that will be this transition’s true ‘Nirvana’.

## Figures and Tables

**Figure 1:**
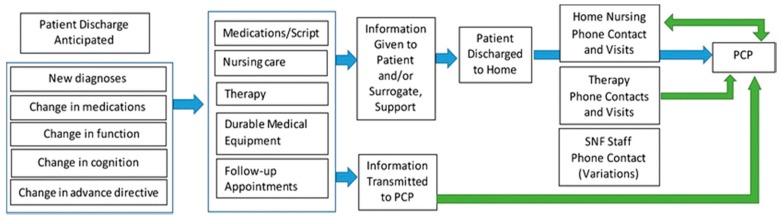
Consensus panel-determined work-flow for a patient transitioning from the skilled nursing facility (SNF) to home.
